# Mechanism of Sphingosine 1-Phosphate- and Lysophosphatidic Acid-Induced Up-Regulation of Adhesion Molecules and Eosinophil Chemoattractant in Nerve Cells

**DOI:** 10.3390/ijms12053237

**Published:** 2011-05-17

**Authors:** Richard W. Costello, Michael Maloney, Mazin Atiyeh, Gerald Gleich, Marie-Therese Walsh

**Affiliations:** 1 Department of Medicine, Royal College of Surgeons in Ireland, Beaumont Hospital, Dublin 9, Ireland; E-Mails: rcostello@rcsi.ie (R.W.C.); micmaloney@rcsi.ie (M.M.); matiyeh@rcsi.ie (M.A.); 2 Department of Dermatology, University of Utah, Salt Lake City, UT 84132, USA; E-Mail: Gerald.Gleich@hsc.utah.edu

**Keywords:** lysophospholipid, neuronal, eosinophil

## Abstract

The lysophospholipids sphingosine 1-phosphate (S1P) and lysophosphatidic acid (LPA) act via G-protein coupled receptors S1P_1–5_ and LPA_1–3_ respectively, and are implicated in allergy. Eosinophils accumulate at innervating cholinergic nerves in asthma and adhere to nerve cells via intercellular adhesion molecule-1 (ICAM-1). IMR-32 neuroblastoma cells were used as an *in vitro* cholinergic nerve cell model. The G_i_ coupled receptors S1P_1_, S1P_3_, LPA_1_, LPA_2_ and LPA_3_ were expressed on IMR-32 cells. Both S1P and LPA induced ERK phosphorylation and ERK- and G_i_-dependent up-regulation of ICAM-1 expression, with differing time courses. LPA also induced ERK- and G_i_-dependent up-regulation of the eosinophil chemoattractant, CCL-26. The eosinophil granule protein eosinophil peroxidase (EPO) induced ERK-dependent up-regulation of transcription of S1P_1_, LPA_1_, LPA_2_ and LPA_3_, providing the situation whereby eosinophil granule proteins may enhance S1P- and/or LPA- induced eosinophil accumulation at nerve cells in allergic conditions.

## 1. Introduction

In animal models of asthma, and in many asthma patients, antigen inhalation results in inhibition of muscarinic M_2_ receptors on parasympathetic nerves, leading to acetylcholine release and hence mucous secretion and contraction of bronchial smooth muscle [1].

Eosinophilia is a hallmark of allergic diseases such as asthma and allergic rhinitis [2]. Eosinophils accumulate at innervating cholinergic nerves in human and animal models of allergic conditions, contributing to nerve hyperresponsiveness due to eosinophil major basic protein (MBP)-mediated M_2_ antagonism [3,4]. Eosinophil accumulation at airway nerves is mediated by eosinophil CCR3 receptors [5]. We have employed the neuroblastoma cell line IMR-32 as a useful *in vitro* model of cholinergic nerve cell function. Differentiated IMR-32 cells express neuronal muscarinic M_2_ receptors and release acetylcholine in response to eosinophil adhesion via nerve intercellular adhesion molecule-1 (ICAM-1) and vascular cell adhesion molecule-1 (VCAM-1) [6–8]. Adhesion results in both the release of eosinophil granule proteins and the generation of signaling events and phenotypic changes within the nerve cells [6,9–13]. Released eosinophil granule proteins [6] induce further nerve cell signaling and protection from apoptosis [12]. Thus, eosinophils influence nerve function due to direct contact via ICAM-1 and VCAM-1 and consequent release of granule proteins.

S1P and LPA are bioactive phospholipids, which exert many of their effects via G-protein coupled S1P receptors S1P_1_, S1P_2_, S1P_3_, S1P_4_ and S1P_5_ and LPA receptors LPA_1_, LPA_2_, LPA_3_ and the structurally distant LPA_4_ respectively [14–16] as well as LPA5 which has a distinct expression pattern suggesting specific physiological functions [17]. S1P and LPA have both been implicated in various aspects of neuronal function and activity and neuronal cell intracellular signaling, including cytoskeletal reorganization, acetylcoholine release, apoptosis and neurite outgrowth or retraction [18–21].

A role for both S1P and LPA and their receptors has also been postulated in airway inflammatory responses, for example in allergy (reviewed [22,23]. Both S1P and LPA levels in bronchoalveolar lavage fluid are significantly increased 18–24 h following segmental allergen challenge in allergy patients and S1P promotes airway remodeling [24,25]. S1P is released primarily from activated platelets and activated mast cells [26]. S1P promotes airway remodeling [24] and we and others have shown that eosinophils express functional S1P receptors [27,28]. *In vitro* and animal studies have indicated that LPA induces eosinophil and neutrophil migration [29–31]. LPA also induces mast cell chemokine and histamine release [32,33]. Both S1P and LPA up-regulate intercellular adhesion molecule-1 (ICAM-1) in HUVECs [34]. Importantly in the context of allergy, S1P up-regulates ICAM-1 in human alveolar epithelial cells [35–37].

We hypothesized that cholinergic nerve cells would express receptors for S1P and LPA and that ligand binding to these receptors would activate intracellular signaling pathways in the nerve cells leading to transcriptional up-regulation of ICAM-1 and of members of the eosinophil eotaxin chemoattractant family. This would further implicate S1P and/or LPA in allergic symptoms by enhancing eosinophil accumulation at and adhesion to nerve cells and hence to nerve cell hyperreactivity.

## 2. Results and Discussion

### 2.1. IMR32 Cells Express S1P_1_, S1P_3_ and LPA_1–3_; S1P and LPA Induce Up-Regulation of ICAM-1 Transcription

Given that there is both an increased level and defined source of both LPA and SIP after allergen challenge we investigated the role of these lysophospholipids in an *in vitro* model of eosinophil/nerve cell interactions. We and others have used butyrate-differentiated IMR-32 cells as a model of cholinergic nerve function in a variety of studies [6–11,13,38,39]. They are considered to be a convenient *in vitro* model for studying aspects of neurobiology at the molecular and cell level.

**Figure 1.**
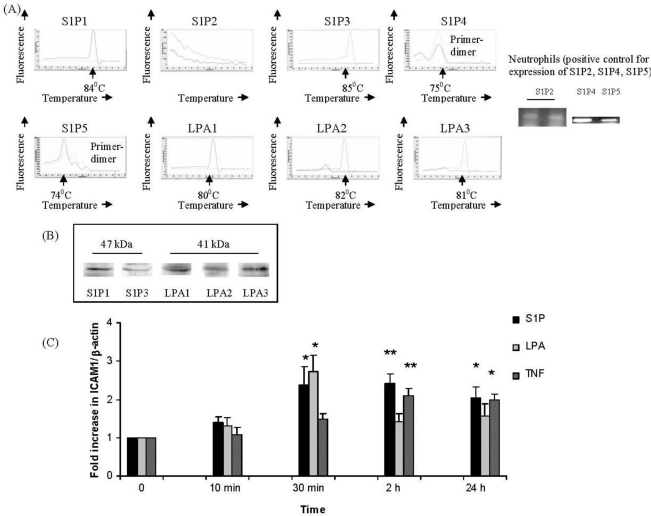
S1P and LPA receptors in IMR-32 cells and S1P- or LPA- induced ICAM-1 transcription. IMR32 cells were plated in 6-well tissue culture dishes in differentiation medium (5 × 10^5^ per well) then harvested for: (**A**) RNA and cDNA preparation and semi-quantitative or quantitative PCR using primers for (**A**) S1P_1–5_ or LPA_1–3_ or (**B**) total protein preparation and Western blotting for receptors S1P_1_, S1P_3_ or LPA_1–3_. (**C**) IMR32 cells in differentiation medium were treated with: S1P (1 μM), LPA (1 μM) or TNF (10 ng/mL) for the indicated times, then harvested for RNA and cDNA preparation and real-time PCR using primers for ICAM-1 or β-actin. Results are expressed as fold increase in ICAM-1/β-actin ratio over non-EPO treated (0) cells (set to unity). Mean ± SEM; * *p* < 0.05, **p < 0.01, significantly increased *versus* untreated cells.

Expression of S1P receptors S1P_1_ and S1P_3_ (Figure 1A) and of LPA_1–3_ (Figure 1B) was confirmed in IMR32 cells maintained in proliferation or differentiation medium by real-time PCR. No expression of S1P_2,_ S1P_4_ or S1P_5_ was detected after 40 cycles of PCR in any cells (Figure 1A); as a positive control, expression of all three of these receptors was readily detected on neutrophils (Figure 1A). All further experiments were in differentiating IMR32 cells, as these cells display a strongly cholinergic phenotype in differentiation medium [8,24,38]. We confirmed by Western blotting that, identically to the cDNA expression profile, differentiating IMR32 cells expressed S1P_1_, S1P_3_ (Figure 1B) and LPA_1–3_ proteins (Figure 1B), but not S1P_2_, S1P_4_ or S1P_5_. We determined by real time PCR that both S1P (Figure 1C) and LPA (Figure 1C) increased expression of ICAM-1 two- to three-fold over baseline levels, a similar order of magnitude as the positive control stimulus, TNF- (Figure 1C). However, up-regulation of ICAM-1 expression was induced more transiently by LPA (Figure 1C), compared to S1P which induced a sustained up-regulation between 30 min to 24 h (Figure 1C), similar to TNF- (Figure 1C).

### 2.2. S1P and LPA Induce ERK Activation in IMR32 Cells with Different Time Courses with Consequenct G_i_-Protein Coupled and ERK-Dependent Up-Regulation of ICAM-1 and/or CCL-26

**Figure 2.**
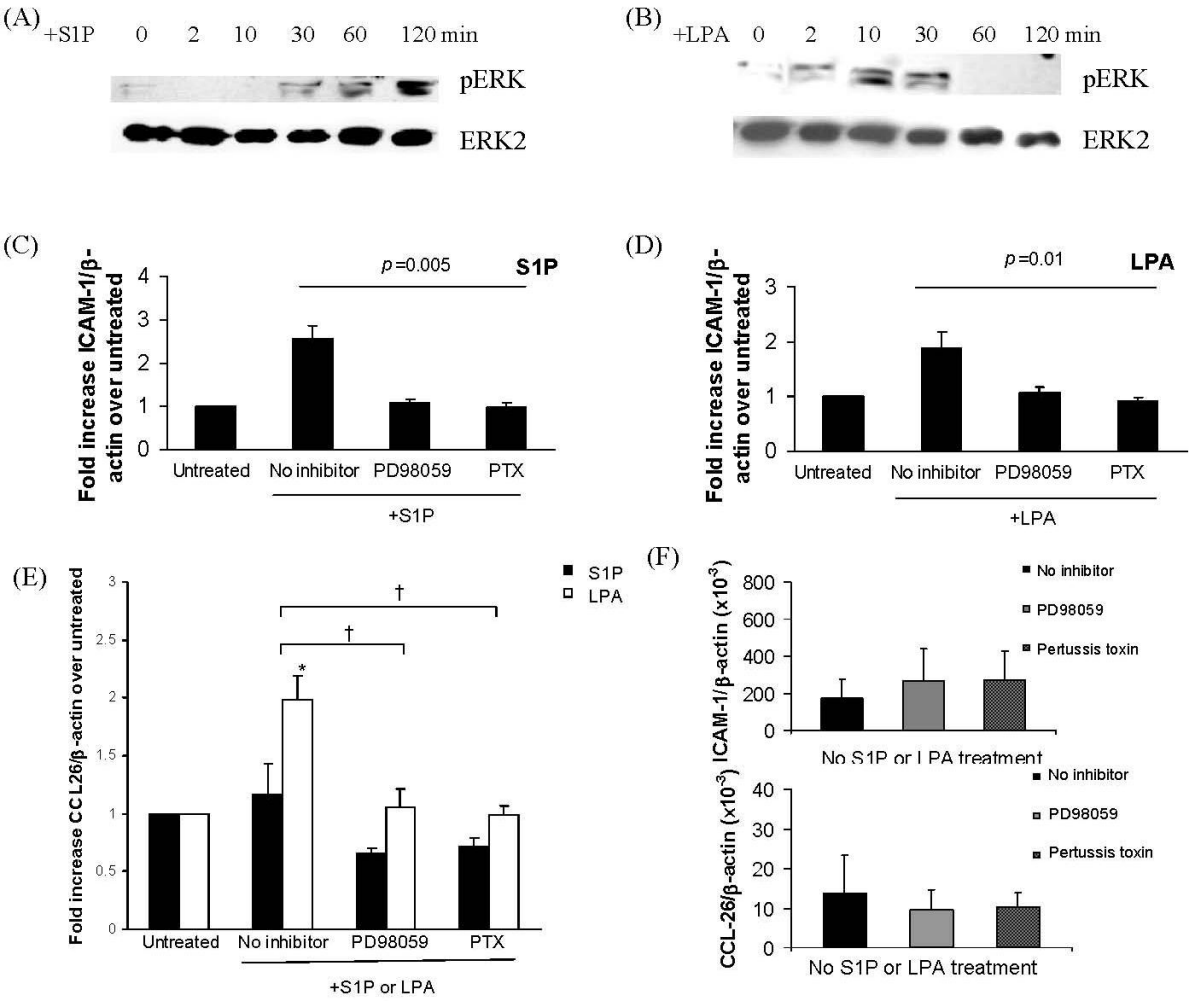
S1P and LPA induce G_i_-mediated ERK phosphorylation and differential up-regulation of ICAM-1 and CCL-26. IMR32 cells in differentiation medium were treated with: S1P (**A**, **C**, **E**) (1 μM) or LPA (1 μM) (**B**, **D**, **E**) for the indicated times. Cells were harvested for cytoplasmic protein and subjected to Western blotting with antibody to phosphor-ERK (A, B, top panels) then ERK2 (A, B, bottom panels) for normalization Blots shown are representative of three similar experiments. Real-time PCR was carried out on cDNA from cells stimulated with S1P or LPA (both 1 μM) in the presence or absence of pre-treatment overnight with the MEK/ERK inhibitor PD98059 (50 μM) or the G_i_ protein coupling inhibitor, pertussis toxin (PTX) (0.1 μg/mL) (**C**, **D**, **E**), using primers for ICAM-1 (**C**, **D**) or CCL-26 (**E**) or β-actin (**C**, **D**, **E**). Results are expressed as fold increase in ICAM-1/β-actin or CCL-26/β-actin ratio over untreated cells. Mean ± SEM, *n* = 4; * *p* < 0.05, LPA-induced fold increase over untreated; ^†^ *p* < 0.05, PD98059 or PTX-induced reduction in LPA-mediated CCL26 up-regulation. Panel F shows that the baseline absolute ratio of ICAM-1 or CCL-26 to β-actin is not significantly different in the presence of either inhibitor pre-treatment.

To determine the intracellular signaling mechanisms that could mediate S1P- and/or LPA-induced up-regulation of ICAM-1 on IMR32 cells, activation of the ERK MAP kinase was examined. Both S1P (Figure 2A) and LPA (Figure 2B) induced ERK phosphorylation but with different time courses. S1P induced activation of ERK was observable at 30 min of stimulation and remained robust at 1–2 h (Figure 2A) while LPA induced a more rapid ERK activation which had fallen to baseline levels between 30 and 60 min of stimulation (Figure 2B). To further determine intracellular mediators involved in S1P- and LPA-induced ICAM-1 up-regulation, IMR32 cells were incubated with S1P (1 μM, 2 h) or LPA (1 μM, 30 min). These treatment times were chosen based on the time course for ICAM-1 induction (Figure 1C) which suggested that these were the optimal times for S1P and LPA respectively. Cells were pre-treated with either the MEK inhibitor PD98059, which prevents ERK phosphorylation or with pertussis toxin (PTX), to inhibit the G_i_ protein, to which all the receptors S1P_1_, S1P_3_ and LPA_1–3_ can couple. S1P-induced (Figure 2C) or LPA-induced (Figure 2D) up-regulation of ICAM-1 was measured by real-time PCR and compared between cells pre-treated with PD98059 or PTX and non-pre-treated cells. This revealed that up-regulation of ICAM-1 by both S1P and by LPA was dependent on both ERK phosphorylation and on G_i_ coupling. This is consistent with the recently demonstrated mechanism of S1P-induced up-regulation of ICAM-1 in airway epithelial cells [34].

Real-time PCR was also used to monitor expression of the eosinophil chemoattractant CCL26 (eotaxin-3), normalized to β-actin expression (Figure 2E). Results indicated that at the time points shown, LPA but not S1P up-regulated CCL26 expression (Figure 2E); we have also observed no up-regulation of CCL26 by S1P at other time points (data not shown). LPA-induced CCL26 up-regulation was dependent on ERK phosphorylation and on G_i_ coupling (Figure 2E). This is the first time that LPA but not S1P has been shown to up-regulate expression of an eosinophil chemoattractant, CCL26. These results imply that further intracellular mediators are involved in CCL26 expression, which are activated by LPA but not S1P, for example the transcription factor STAT-6 [40].

Our previous studies have shown that eosinophils accumulate at cholinergic nerves in human and animal models of allergic conditions such as asthma and rhinitis [41]. *In vitro*, eosinophils adhere to primary nerve cells and to differentiated IMR-32 cells via the adhesion molecules ICAM-1 and VCAM-1, with implications for nerve cell cholinergic phenotype and survival [8–13,38]. In a guinea pig model of asthma, accumulation of eosinophils at nerves was significantly decreased by treatment with CCR3 antagonist prior to antigen inhalation [5]. CCR3 antagonism also consequently reduced antigen-associated loss of M_2_ receptor function [5]. LPA induced up-regulation of expression of CCL26 message in differentiated IMR-32 cells; CCL26 (eotaxin-3) is a CCR3 agonist, which is up-regulated in asthmatics following allergen challenge [42] CCL26 rather than CCL11 (eotaxin-1) or CCL24 (eotaxin-2) is therefore suggested to be responsible for continuing eosinophil recruitment at later stages following allergen challenge. Furthermore, both S1P and LPA induced up-regulation of ICAM-1. Thus S1P and LPA induce conditions whereby eosinophil migration and adhesion to nerve cells would be favoured. In the context of allergy, this suggests a previously unrecognized role for these lysophospholipids in promotion of eosinophil interaction with nerves and hence nerve cell hyperreactivity, with its associated bronchoconstriction.

### 2.3. The Eosinophil Granule Protein EPO Induces Up-Regulation of S1P_1_, LPA_1_, LPA_2_ and LPA_3_ Receptors Expression

**Figure 3.**
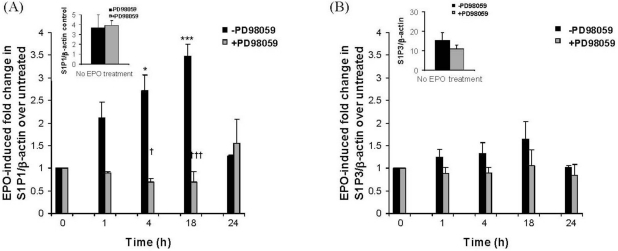
Eosinophil peroxidase induces ERK-dependent transcriptional up-regulation of S1P_1_ but not S1P_3_ in IMR32 cells. IMR32 cells in differentiation medium were pre-treated or not overnight with PD98059 (50 μM). Cells were then treated with eosinophil peroxidase (EPO) (1 μg/mL) for the indicated times then harvested for RNA and cDNA preparation and real-time PCR using primers for: (**A**) S1P_1_ or (B) S1P_3_ or β-actin (**A** and **B**). Results are expressed as EPO-induced fold increase in S1P receptor/β-actin ratio over non-EPO treated cells harvested at the same time as the 24 hour time point, which are set to unity. Inserts show absolute values of S1P receptor *versus* β-actin in non-EPO-treated cells, cultured in the presence or absence of PD98059 pre-treatment, and harvested at the same time as the 24 h time point cells. Mean ± SEM; * *p* < 0.05, *** *p* < 0.001, EPO-induced fold increase in S1P_1_ over untreated; † *p* < 0.05, ††† *p* < 0.001, PD98059-induced reduction in EPO-mediated S1P_1_ up-regulation.

**Figure 4.**
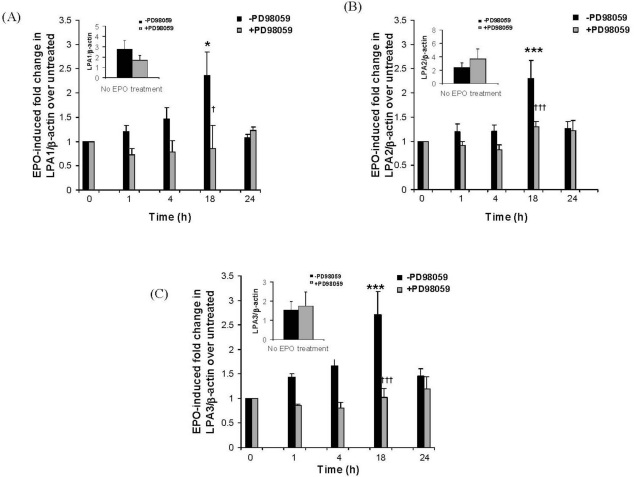
Eosinophil peroxidase induces ERK-dependent transcriptional up-regulation of LPA_1_, LPA_2_ and LPA_3_ in IMR32 cells. IMR32 cells in differentiation medium were pre-treated or not overnight with PD98059 (50 M). Cells were then treated with eosinophil peroxidase (EPO) (1 μg/mL) for the indicated times then harvested for RNA and cDNA preparation and real-time PCR using primers for: (**A**) LPA_1_ or (**B**) LPA_2_ or (**C**) LPA_3_ or β-actin (**A**, **B** and **C**). Results are expressed as EPO-induced fold increase in LPA receptor/β-actin ratio over non-EPO treated cells harvested at the same time as the 24 h time point, which are set to unity. Inserts show baseline absolute values of LPA receptor *versus* β-actin in non-EPO treated cells, cultured in the presence or absence of PD98059 pre-treatment, and harvested at the same time as the 24 hour time point cells. Mean ± SEM; * *p* < 0.05, *** *p* < 0.001, EPO-induced fold increase in LPA receptor over untreated; † *p* < 0.05, ^†††^ *p* < 0.001, PD98059-induced reduction in EPO-mediated S1P_1_ up-regulation.

We have previously shown that eosinophil adhesion to IMR32 cells induces eosinophil degranulation and release of granule proteins, including eosinophil peroxidase (EPO) [8–10]. To determine whether released eosinophil granule protein could increase expression of IMR32 S1P or LPA receptors, nerve cells were treated with eosinophil peroxidase (EPO) (1 μg/mL) for 1, 4, 18 or 24 h in the presence or absence of PD98059. Real-time PCR was used to monitor the expression of S1P_1_ and S1P_3_ receptors (Figure 3) or LPA_1_, LPA_2_ and LPA_3_ receptors (Figure 4). EPO significantly enhanced expression of S1P_1_ by approximately 3–4 fold between 4–18 h of stimulation (Figure 3A). This up-regulation was dependent on ERK activation as it was abolished in the presence of PD98059. S1P_3_ expression was unaffected by EPO (Figure 3B). LPA_1_ (Figure 4A), LPA_2_ (Figure 4B) and LPA_3_ (Figure 4C) expression were all raised approximately 3-fold at 18 h of stimulation, then fell back to control levels by 24 h.

These results imply that S1P and LPA, acting via their nerve cell receptors, can promote eosinophil recruitment and adhesion and that subsequently, eosinophil adhesion and the resulting degranulation can release a factor, namely EPO, which up-regulates the LPA and S1P receptors responsible for this effect and hence perpetuate or prolong it.

In light of the results of our study, it would be of interest to determine whether antagonism of individual S1P and/or LPA receptors reduces accumulation at and/or adhesion of eosinophils to nerves in animal models of asthma, similarly to CCR3 antagonism. Up-regulation of ICAM-1 by both S1P and LPA was dependent on G_i_-coupling, as was LPA-induced up-regulation of CCL26. All the receptors IMR-32-expressed receptors, S1P_1_, S1P_3_, LPA_1_, LPA_2_ and LPA_3_, couple to G_i_. Definitive receptor identification awaits further studies utilizing receptor-specific ligands or multiple siRNA technology, which was considered impractical for this work given the abundance of the specific receptors.

## 3. Experimental Section

*IMR32 nerve cell culture*: The human cholinergic neuroblastoma cell line IMR32 was depleted of fibroblasts, as described previously [10]. The cells were maintained in culture in proliferation medium (DMEM Plus Glutamax, 5% FCS, 100 U/mL penicillin/streptomycin; GIBCO^®^ Invitrogen, Paisley, UK) at 37 °C in an atmosphere of 5% CO_2_. On achieving confluence, cells were plated at a density of 5 × 10^5^/well in 6-well cell culture dishes and grown in proliferation medium for 48 h. Proliferation media was then replaced by differentiation medium (DMEM Plus Glutamax, 2% FCS, 2 mM sodium butyrate (Sigma, Poole, UK), 100 U/mL penicillin/streptomycin) and cells were used for experimentation after a further 6 days. In some experiments, individual wells were maintained in proliferation medium in parallel with the differentiating cells.

*mRNA Analysis*: Total RNA was isolated from the cells with TRI reagent™ (Sigma), according to the manufacturer’s instructions. For both quantitative LightCycler^™^ PCRs (Roche, Mannheim, Germany) and semiquantitative RT-PCRs, 1 μg of RNA was reverse transcribed with AMV reverse transcriptase and oligo-dT primer using a 1st strand cDNA synthesis kit (Roche), according to the manufacturer’s instructions.

Semi-quantitative RT-PCR analysis of cDNA preparations was carried out in 50 μL reactions with Taq-DNA polymerase for S1P_1–5_ or LPA_1–3_, using the primers sets (MWG Biotech/Eurofins AG, Ebersberg, Germany) outlined in Table 1. PCR conditions were: 94 °C, 4 min (1 cycle); 94 °C, 1.5 min, 54 °C, 1.5 min, 72 °C, 2 min (25–40 cycles); 72 °C, 10 min (1 cycle). PCR products were separated by 1.5% agarose gel electrophoresis and photographed under UV illumination. 40 cycles of PCR were carried out to verify absence of S1P_2_, S1P_4_ and S1P_5_.

Quantitative RT-PCR analysis was carried out on the LightCycler^™^ (Roche) using fast start Taq DNA polymerase containing the double-stranded DNA binding dye SYBR Green 1 as in [38]. Primers are shown in Table 1. The samples were denatured at 95 °C for 15 min followed by 35–40 cycles of denaturation, annealing and extension at 95 °C for 15 s, 55 °C for 25 s, and 72 °C for 11 s (ICAM-1, CCL26, S1P_1_, S1P_2_, S1P_3_, S1P_4_, S1P_5_, LPA_1_, LPA_2_, LPA_3_), or 95 °C for 15 s, 55 °C for 20 s, and 72 °C for 20 s (-actin). Characteristic melting curves were obtained at the end of amplification by cooling the samples to 65 °C for 15 s followed by further cooling to 40 °C for 30 s. Serial 10-fold dilutions were prepared from individual PCR products, which were then used as standards to plot against the unknown samples. Quantification of data was analyzed using the LightCycler^™^ analysis software, and values were normalized to the level of β-actin expression for each sample on the same template cDNA. Results for induction over baseline values of ICAM-1 or CCL26 expression by S1P (1 μM) or LPA (1 μM) were obtained; in some experiments fold induction was compared between untreated, differentiated IMR32 cells *versus* cells pre-treated overnight with the MEK/ERK inhibitor PD98059 (Sigma) (50 μM) or with pertussis toxin (Sigma) (0.1 μg/mL). Results for induction over baseline values of S1P_1_, S1P_3_, LPA_1_, LPA_2_ or LPA_3_ by EPO (1 μg/mL, various time points) were also compared between untreated, differentiated IMR32 cells *versus* cells pre-treated overnight with the MEK/ERK inhibitor PD98059 (50 μM).

*Nuclear and cytoplasmic protein preparation*: IMR-32 cells (5 × 10^5^) were differentiated for 6 days with sodium butyrate as described above and then incubated with LPA or S1P for various time periods from 2 min to 2 h. Nuclear and cytoplasmic extracts were isolated from IMR-32 cells, essentially as in [10]. Protein concentration was established by the Bradford method [43] and nuclear and cytoplasmic extracts stored at −80 °C.

*Western blotting:* Total cell protein was extracted from IMR32 cells using Tri Reagent, according to the manufacturer’s instructions and quantified according to the Bradford method [43]. Total cell protein (S1P_1–5_, LPA_1–3_ probing) or cytoplasmic protein (ERK probing) (10 μg) was subjected to Western blotting as previously described [38] Membranes were incubated in blocking buffer (Dulbecco’s PBS (Invitrogen) containing 0.2% (w/v) I-block and 0.1% (v/v) Tween-20) for 1 h at room temperature then incubated for 2 h in blocking buffer containing the individual respective HRP-conjugated primary antibody (Santa Cruz, CA, USA) (1:500 for each). Following six 5-min washes in washing buffer (PBS pH 7.4, 0.1% (v/v) Tween-20) membranes were incubated for 1 h in blocking buffer containing a dilution of the appropriate secondary antibody (1:10,000 dilution). Membranes were then washed 6 times for 5 min each and exposed to SuperSignal West Pico Chemiluminescent Substrate (Pierce Technology Inc., Rockford, IL, USA) for Western Blotting for 5 min at room temperature. Blots were then exposed to X-OMAT light sensitive film to obtain an image.

*Statistical analysis*: Values are expressed as mean SEM. The statistical significance of differences between proliferating and differentiating cells was evaluated by two-tail Student’s *t*-test or by ANOVA, as appropriate. The Graphpad Instat program was used. A *p*-value of 0.05 or less was taken as significant.

## 4. Conclusion

Eosinophils accumulate at cholinergic nerves at sites of inflammation in asthma and allergic rhinitis. We have previously shown that eosinophils adhere to nerve cells via ICAM-1 [6,7]. In this study we have shown that the lysophospholipid LPA induces transcriptional up-regulation of expression of the eosinophil chemoattractant CCL26, which is up-regulated in asthmatics following allergen challenge [42] and is suggested to be responsible for continuing eosinophil recruitment at later stages following allergen challenge. Furthermore, both LPA and another lysophospholipid, S1P, induce up-regulation of ICAM-1. We and others have previously shown that S1P can also induce a direct chemoattractive effect on eosinophils [27,28]. Thus both S1P and LPA induce conditions whereby eosinophil migration and adhesion to nerve cells would be favored. In the context of allergy, this suggests a previously unrecognized role for these lysophospholipids in promotion of eosinophil interaction with nerves and hence nerve cell hyperreactivity, with its associated bronchoconstriction. Furthermore, the eosinophil granule protein EPO, induced up-regulation of the S1P receptor S1P_1_ and the LPA receptors LPA_1–3_. We have previously shown EPO to be released from eosinophils upon their adhesion to nerve cells via ICAM-1. Hence, the potential exists for a perpetuation of the S1P and LPA driven effects on eosinophil and nerve interaction as the EPO released from eosinophils attached to nerve cells could cause up-regulation of the S1P and LPA receptors, hence resulting in more S1P and LPA binding, more up-regulation of ICAM-1 and CCL-26 and more eosinophil accumulation.

## Figures and Tables

**Table 1.** Primers used in this study.

**Gene**	**Primer Sequence**	**Amplicon Length (Base Pairs)**
S1P1	5′-ATCGTCCTGAGCGTCTTCAT-3′ (forward) 5′-CCAGGAAGTACTCCGCTCTG-3′ (reverse)	95
S1P2	5′-CCAAGCATTATGTGCTGTGC-3′ (forward) 5′-CAGAAGGAGGATGCTGAAGG-3′ (reverse)	186
S1P3	5′-ACCAGTACGTGGGGAAGTTG-3′ (forward) 5′-GGCAATCAAAACCATCAGGT-3′ (reverse)	105
S1P4	5′-CCAAGCGCTACATCCTCTTC-3 (forward) 5′-CAGAGGTTGGAGCCAAAGAC-3′ (reverse)	221
S1P5	5′-ACAACTACACCGGCAAGCTC-3′ (forward) 5′-GCCCCGACAGTAGGATGTT-3′ (reverse)	218
LPA1	5′-ATTTCACAGCCCCAGTTCAC-3′ (forward) 5′-CACCAGCTTGCTGACTGTGT-3′ (reverse)	106
LPA2	5′-CTGCTCCTGGATGGTTTAGG-3′ (forward) 5′-CTCGGCAAGAGTACACAGCA-3′ (reverse)	95
LPA3	5′-TTGCCTCTGCAACATCTCTG-3′ (forward) 5′-ATGATGAGGAAGGCCATGAG-3 (reverse)	82
ICAM-1	5′-CAAGGCCTCAGTCAGTGTGA-3′ (forward) 5′-CCTCTGGCTTCGTCAGAATC-3′ (reverse)	129
CCL26	5′-CCTCCTGAGTCTCCACCTTG-3′ (forward) 5′-TGGGAGCAGCTGTTACTGGT-3′ (reverse)	115
-actin	5′-GGACTTCGAGCAAGAGATGG-3′ (forward) 5′-AGGAAGGAAGGCTGGAAGAG-3′ (reverse)	118
